# Supplementary material for: Contrasting suitability and ambition in regional carbon mitigation
Source: Nat Commun. 2022 Jul 14;13:4077. doi: 10.1038/s41467-022-31729-y (PMC9283498; doi:10.1038/s41467-022-31729-y)
Supplement: Supplementary file 1 — Supplementary Information [file 41467_2022_31729_MOESM1_ESM.pdf]

Supplementary Information for

Contrasting suitability and ambition in regional carbon mitigation

Liu et al.

Supplementary Figures

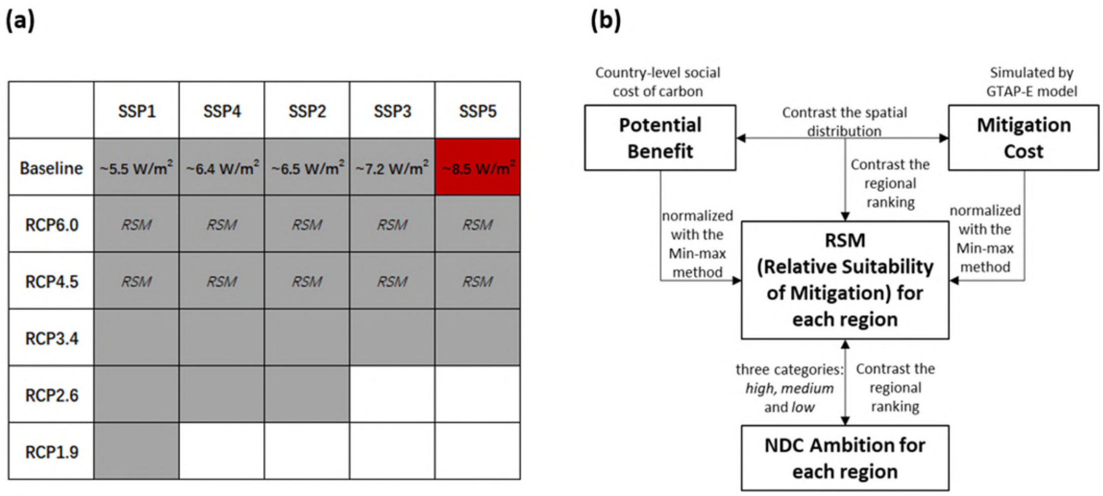

Supplementary Figure 1: Scenarios and framework in this study. (a) A total of 23 scenarios (shown in grey) used to calculate the RCC (average reduction cost of carbon), relative to the reference scenario SSP5-RCP8.5 (SSP5-Baseline, shown in red). The values in the first row indicate the rough amount of radiative forcing under "Baseline" for each SSP. Ten scenarios indicated with "*RSM*" have both RCC and SCC (social cost of carbon) results available to calculate RSM (relative suitability of mitigation). (b) Methodological framework of this study.

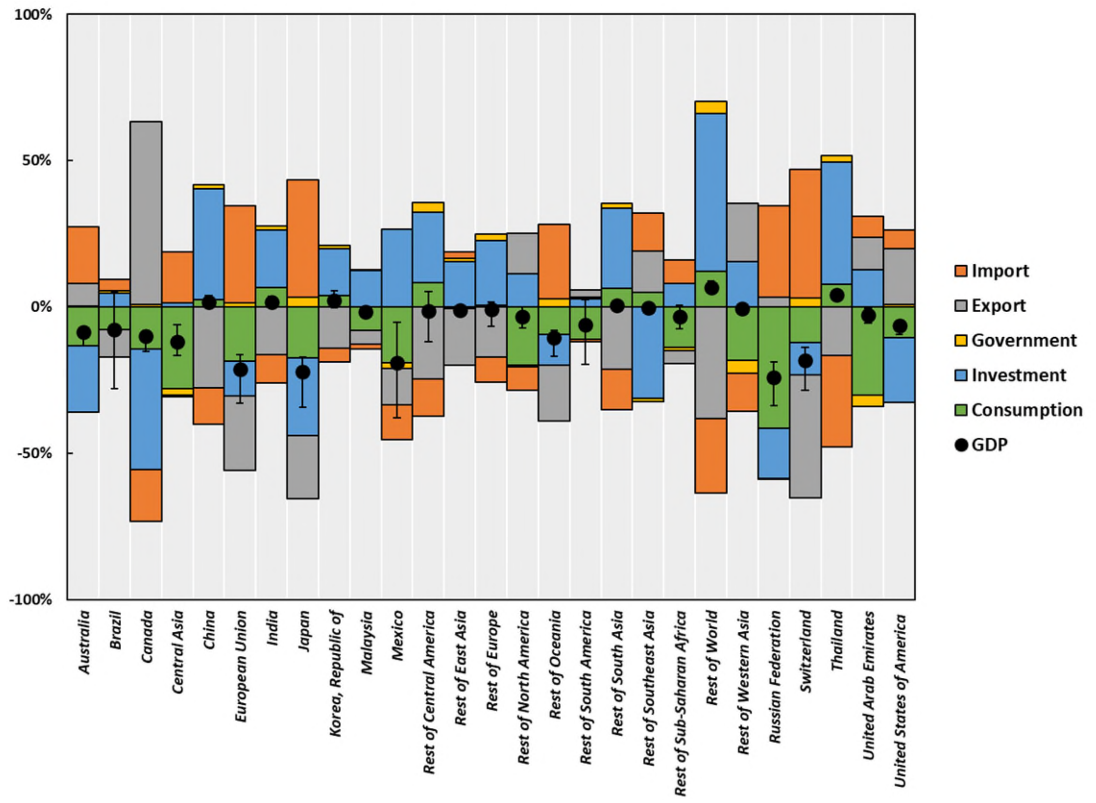

Supplementary Figure 2. Decomposition of GDP change for each region on the expenditure side under SSP2-RCP 4.5. The error bar represents the maximum and minimum GDP change based on results from five IAMs.

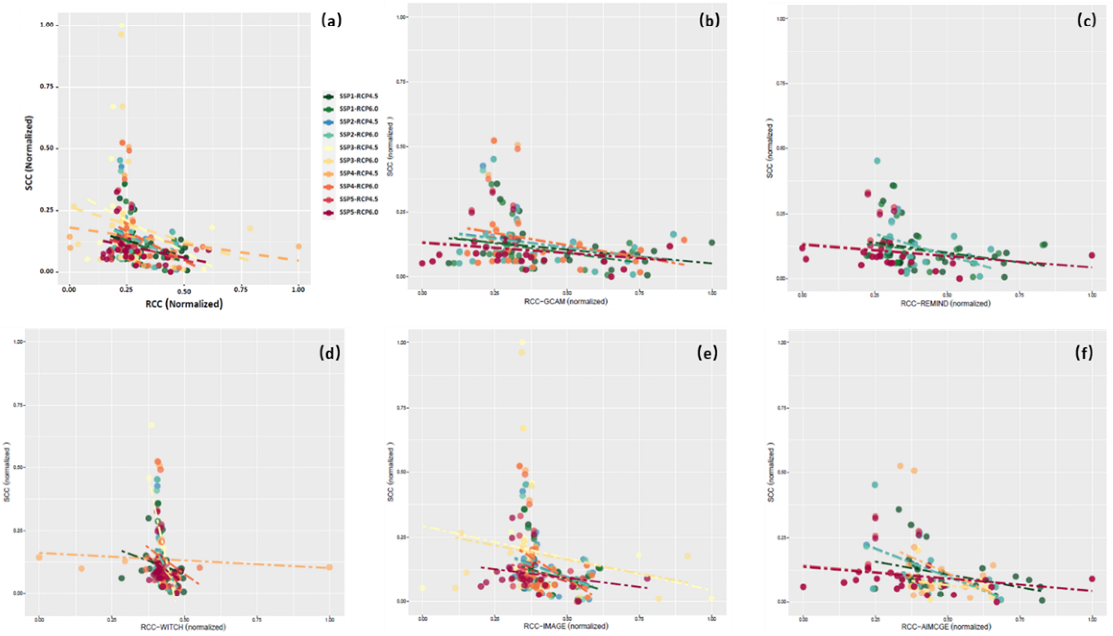

Supplementary Figure 3: Contrasting regional RCC (average reduction cost of carbon) and SCC (social cost of carbon) under each mitigation scenario. (a) Results are averaged over five IAMs. (b-f) Results are based on each IAMs. The dashed lines indicate linear fitting.

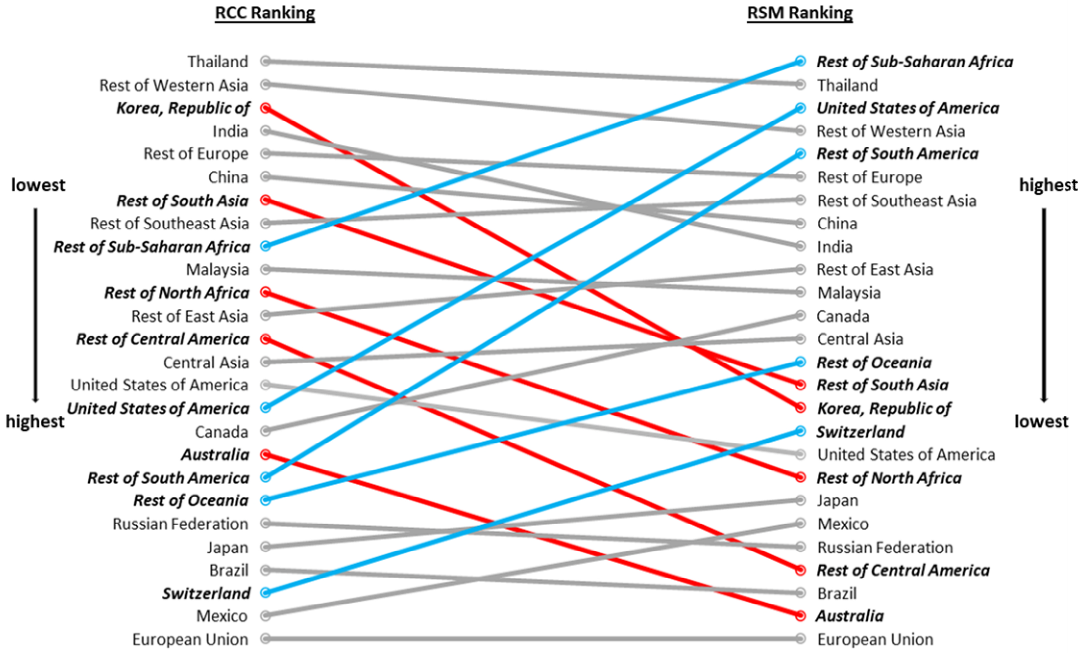

Supplementary Figure 4: Contrasting the regional ranking of RCC (average reduction cost of carbon) and RSM (relative suitability of mitigation) under SSP2-RCP4.5. Regions with gray lines have small differences (within 5) in ranking between RCC and RSM. Regions with blue or red line denote larger differences in ranking between RCC and RSM.

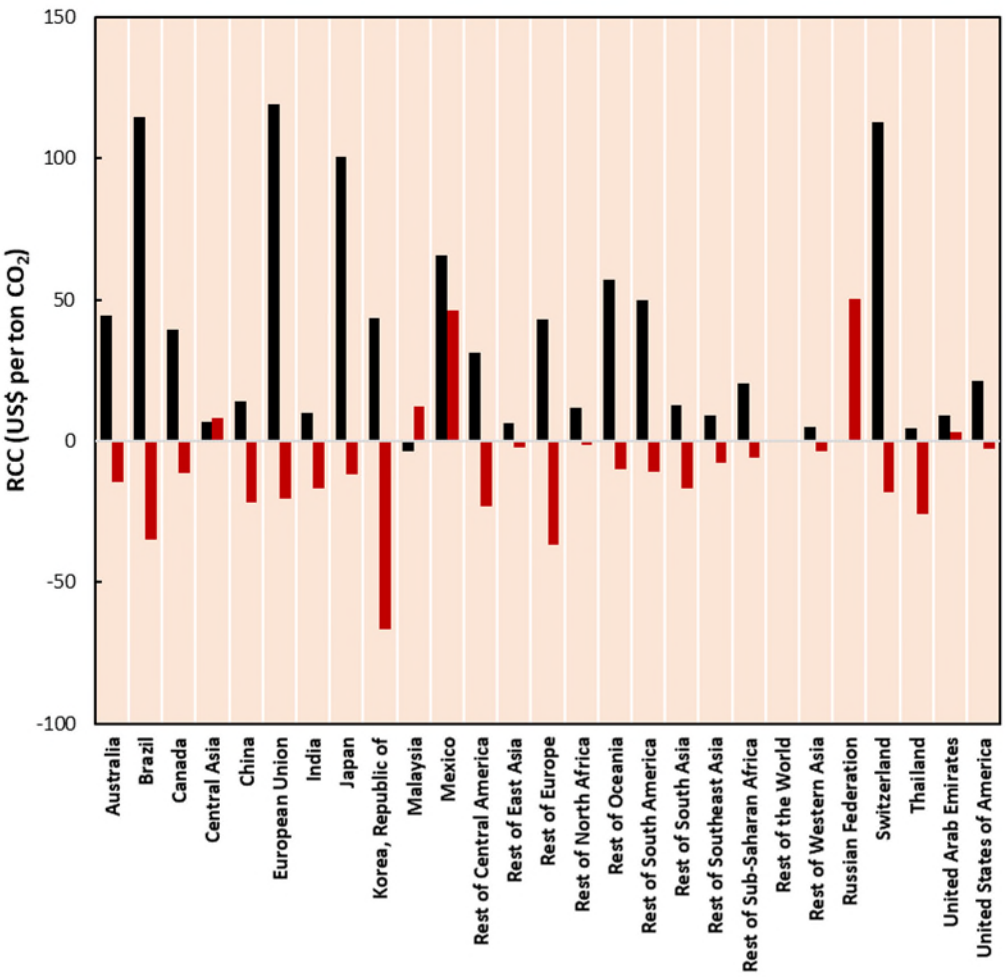

Supplementary Figure 5: The decomposition of RCC (average reduction cost of carbon) for each region under SSP2-RCP4.5. The black bar represents the contribution to RCC from one region itself, and the red bar represents the contributions to RCC from the sum of other regions. More detailed results can be found in Supplementary Data 5.

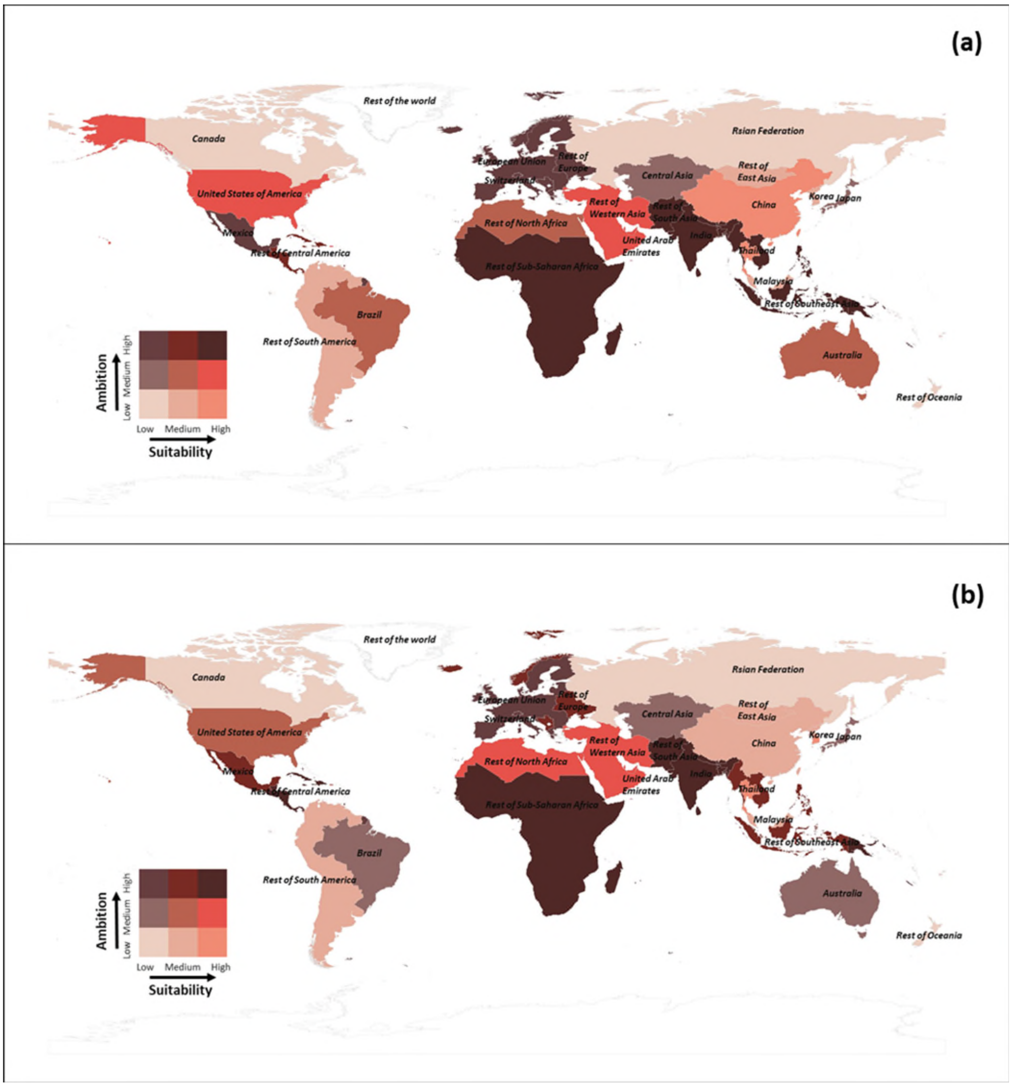

Supplementary Figure 6: Comparison of RSM (relative suitability of mitigation) based on SCC (social cost of carbon) by Ricke et al and Yang et al. (a) Contrasting suitability and ambition of carbon mitigation among 27 emitting countries and regions with RSM constructed based on SCC by Ricke et al. (b) Contrasting suitability and ambition of carbon mitigation among 27 emitting countries and regions with RSM constructed based on SCC by Yang et al.

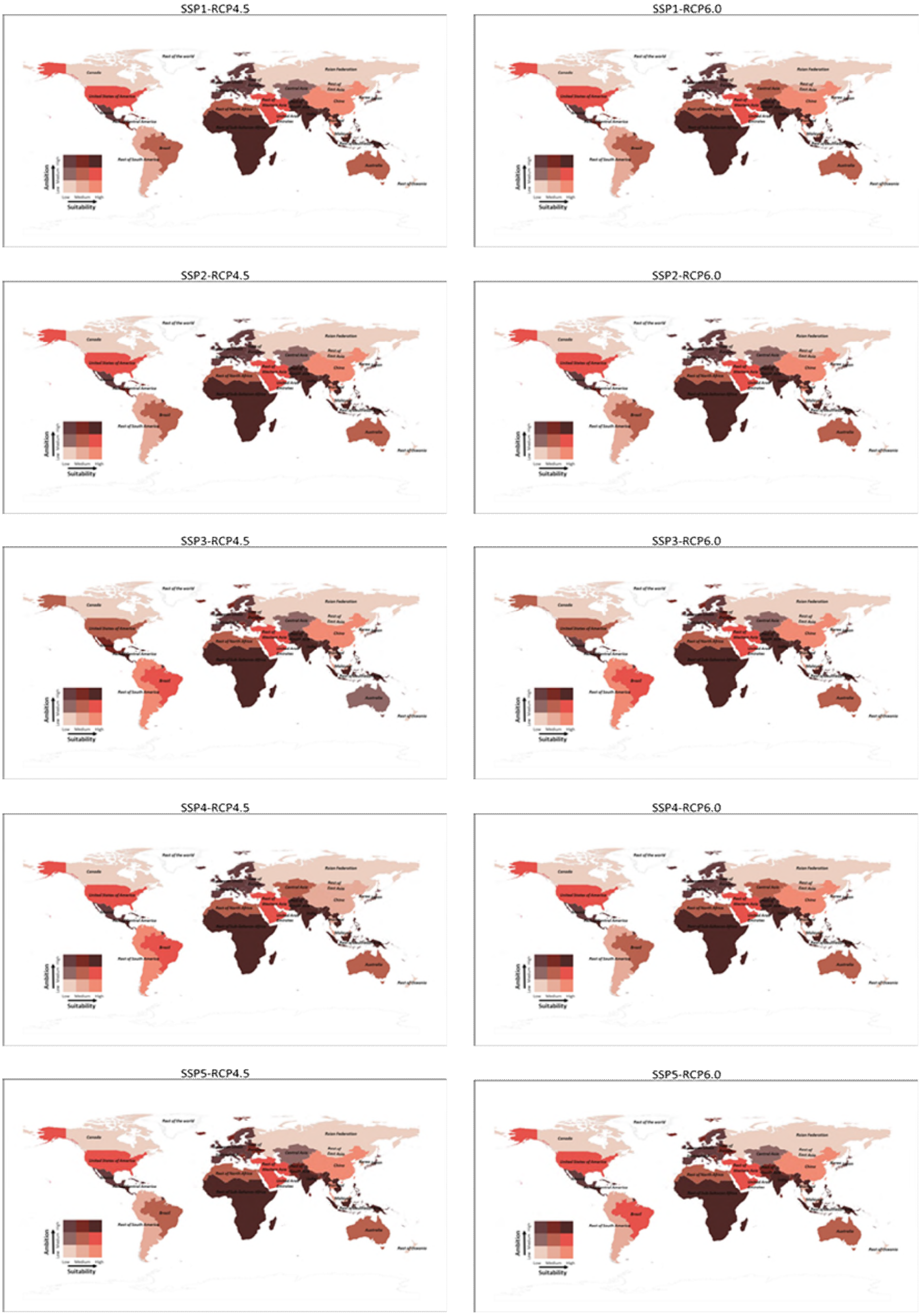

Supplementary Figure 7: Comparison of RSM (relative suitability of mitigation) and NDC (national determined contribution) ambition for each region under each mitigation scenario. For both RSM and ambition scores, “High” represents the top 1/3 among the 27 regions, “Medium” represents the middle 1/3, and “Low” represents the bottom 1/3.
