# Supplementary material for: Contrasting suitability and ambition in regional carbon mitigation
Source: Nat Commun. 2022 Jul 14;13:4077. doi: 10.1038/s41467-022-31729-y (PMC9283498; doi:10.1038/s41467-022-31729-y)
Supplement: Supplementary file 3 — Description of Addtional Supplementary Information [file 41467_2022_31729_MOESM3_ESM.docx]

Title: Supplementary Data 1
Description: Mapping of regions and sectors from GTAP to this study.

Title: Supplementary Data 2
Description: Mitigation target of each region under each scenario.

Title: Supplementary Data 3
Description: Average reduction cost of carbon of each region under each scenario.

Title: Supplementary Data 4
Description: RSM results for each region under each scenario.

Title: Supplementary Data 5
Description: Sensitivity experiments for testing price transmission mechanism in GTAP-E model and testing the robust of simulated results with different kind of SCC.
