# Supplementary material for: Contrasting suitability and ambition in regional carbon mitigation
Source: Nat Commun. 2022 Jul 14;13:4077. doi: 10.1038/s41467-022-31729-y (PMC9283498; doi:10.1038/s41467-022-31729-y)
Supplement: Supplementary file 9 — Reporting Summary [file 41467_2022_31729_MOESM9_ESM.pdf]

nature portfolio

Corresponding author(s): Mingxi Du

Last updated by author(s): Jun 24, 2022

Reporting Summary

Nature Portfolio wishes to improve the reproducibility of the work that we publish. This form provides structure for consistency and transparency in reporting. For further information on Nature Portfolio policies, see our Editorial Policies and the Editorial Policy Checklist.

Statistics

For all statistical analyses, confirm that the following items are present in the figure legend, table legend, main text, or Methods section.

n/a	Confirmed	
x		The exact sample size (n) for each experimental group/condition, given as a discrete number and unit of measurement
x		A statement on whether measurements were taken from distinct samples or whether the same sample was measured repeatedly
x		The statistical test(s) used AND whether they are one- or two-sided<br>*Only common tests should be described solely by name; describe more complex techniques in the Methods section.*
x		A description of all covariates tested
x		A description of any assumptions or corrections, such as tests of normality and adjustment for multiple comparisons
	x	A full description of the statistical parameters including central tendency (e.g. means) or other basic estimates (e.g. regression coefficient) AND variation (e.g. standard deviation) or associated estimates of uncertainty (e.g. confidence intervals)
x		For null hypothesis testing, the test statistic (e.g. F, t, r) with confidence intervals, effect sizes, degrees of freedom and P value noted<br>*Give P values as exact values whenever suitable.*
x		For Bayesian analysis, information on the choice of priors and Markov chain Monte Carlo settings
x		For hierarchical and complex designs, identification of the appropriate level for tests and full reporting of outcomes
x		Estimates of effect sizes (e.g. Cohen's d, Pearson's r), indicating how they were calculated

Our web collection on statistics for biologists contains articles on many of the points above.

Software and code

Policy information about availability of computer code

Data collection	Economic data and emission data are based on the GTAP database (v10a) for 2014.
Data analysis	GTAP-E computable general equilibrium model is used to estimate RCC; ArcGIS (version 10.5), Excel (Microsoft 365MSO) and R (version 3.6.3) are used to do calculations and draw figures.

For manuscripts utilizing custom algorithms or software that are central to the research but not yet described in published literature, software must be made available to editors and reviewers. We strongly encourage code deposition in a community repository (e.g. GitHub). See the Nature Portfolio guidelines for submitting code & software for further information.

Data

Policy information about availability of data

All manuscripts must include a data availability statement. This statement should provide the following information, where applicable:
- Accession codes, unique identifiers, or web links for publicly available datasets
- A description of any restrictions on data availability
- For clinical datasets or third party data, please ensure that the statement adheres to our policy

Source data are provided with this paper. All data used here are cited in the text or provided in the supplementary files.

Human research participants

Policy information about studies involving human research participants and Sex and Gender in Research.

Reporting on sex and gender	This information has not been collected. This information is not relevant here.
Population characteristics	See above. This information is not relevant here.
Recruitment	See above. This information is not relevant here.
Ethics oversight	See above. This information is not relevant here.

Note that full information on the approval of the study protocol must also be provided in the manuscript.

Field-specific reporting

Please select the one below that is the best fit for your research. If you are not sure, read the appropriate sections before making your selection.

☐ Life sciences ☐ Behavioural & social sciences ☒ Ecological, evolutionary & environmental sciences

For a reference copy of the document with all sections, see nature.com/documents/nr-reporting-summary-flat.pdf

Ecological, evolutionary & environmental sciences study design

All studies must disclose on these points even when the disclosure is negative.

Study description	Here we contrast the cost, calculated with a widely used global economic model, and benefit, estimated as avoided social cost of carbon, to quantify each emitter's relative suitability of carbon mitigation (RSM) across 27 major emitting countries and aggregated regions. We then contrast the RSM and ambition of each emitter to reveal opportunities to enhance mitigation ambition and collaborative action.
Research sample	141 countries and regions and 65 sectors based on GTAP database (v10a) are involved in this study.
Sampling strategy	For this study, the 141 countries and regions have been aggregated to 27 regions, which specify major producers, consumers, and importers/exporters. The 65 production sectors are aggregated to a total of 8 sectors.
Data collection	Economic data and emission data are from the GTAP database for 2014; RCC data is estimated by GTAP-E model; SCC data is from Ricke et al; NDC ambiton score is derived from Robiou du Pont et al.
Timing and spatial scale	The RCC for each region is based on economic data and emission data for 2014, and estimated for 2020 and 2030 under multiple developing and mitigating scenarios.
Data exclusions	No data were excluded.
Reproducibility	Source data are provided with this paper. All data used here are cited in the text or provided in the supplementary files; All computer codes generated during this study are available from the corresponding authors upon reasonable request.
Randomization	All countries and regions and sectors are involved in this study. So randomization is not relevant here.
Blinding	This study is based on existing data. So blinding is not relevant here.

Did the study involve field work? ☐ Yes ☒ No

Reporting for specific materials, systems and methods

We require information from authors about some types of materials, experimental systems and methods used in many studies. Here, indicate whether each material, system or method listed is relevant to your study. If you are not sure if a list item applies to your research, read the appropriate section before selecting a response.

Materials & experimental systems

n/a	Involved in the study
☒	☐ Antibodies
☒	☐ Eukaryotic cell lines
☒	☐ Palaeontology and archaeology
☒	☐ Animals and other organisms
☒	☐ Clinical data
☒	☐ Dual use research of concern

Methods

n/a	Involved in the study
☒	☐ ChIP-seq
☒	☐ Flow cytometry
☒	☐ MRI-based neuroimaging
